# Green Extraction of Alkaloids and Polyphenols from *Peumus boldus* Leaves with Natural Deep Eutectic Solvents and Profiling by HPLC-PDA-IT-MS/MS and HPLC-QTOF-MS/MS

**DOI:** 10.3390/plants9020242

**Published:** 2020-02-13

**Authors:** Jeniffer Torres-Vega, Sergio Gómez-Alonso, José Pérez-Navarro, Edgar Pastene-Navarrete

**Affiliations:** 1 Pharmacognosy laboratory, Department of Pharmacy, Faculty of Pharmacy, Unidad de Desarrollo Tecnológico (UDT), University of Concepción, Concepción 4191996, Chile; jeniffertorres@udec.cl; 2 Regional Institute for Applied Scientific Research, Faculty of Chemical Sciences, University of Castilla-La Mancha, Castilla-La Mancha, 10, 1307 Ciudad Real, Spain; Sergio.Gomez@uclm.es (S.G.-A.); Jose.PNavarro@uclm.es (J.P.-N.); 3 Laboratorio de Síntesis y Biotransformación de Productos Naturales, Dpto. Ciencias Básicas, Universidad del Bio-Bio, Chillan 3780000, Chile

**Keywords:** *Peumus boldus*, boldine, mass spectrometry, natural deep eutectic solvents

## Abstract

*Peumus boldus* Mol., is a Chilean medicinal tree used for gastrointestinal and liver diseases. Such medicinal properties are associated with the presence of bioactive flavonoids and aporphine alkaloids. In this study, a new green and efficient extraction method used seven natural deep eutectic solvents (NADES) as extraction media. The extraction efficiency of these NADES was assessed, determining the contents of boldine and total phenolic compounds (TPC). Chemical profiling of *P. boldus* was done by high-performance liquid chromatography coupled to photo diode array detector and electrospray ion-trap mass spectrometry (HPLC-PDA-ESI-IT/MS) and electrospray ionization quadrupole time-of-flight high-resolution mass spectrometry (HPLC-ESI-QTOF-MS). Among the NADES tested, NADES4 (choline chloride-lactic acid) and NADES6 (proline-oxalic acid) enable better extraction of boldine with 0.427 ± 0.018 and 2.362 ± 0.055 mg of boldine g^−1^ of plant, respectively. Extraction of boldine with NADES4 and NADES6 was more efficient than extractions performed with methanol and water. On the other hand, the highest TPC were obtained using NADES6, 179.442 ± 3.79 mg of gallic acid equivalents (GAE g^−1^). Moreover, TPC in extracts obtained with methanol does not show significant differences with NADES6. The HPLC-PAD-MS/MS analysis enable the tentative identification of 9 alkaloids and 22 phenolic compounds. The results of this study demonstrate that NADES are a promising green extraction media to extract *P. boldus* bioactive compounds and could be a valuable alternative to classic organic solvents.

## 1. Introduction

**Figure 1:**
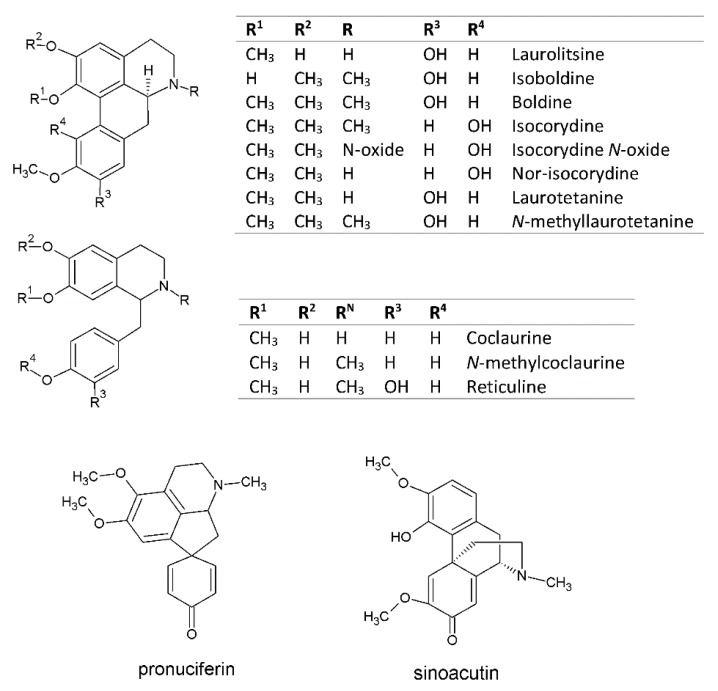
Structure of main alkaloids identified from *Peumus boldus* leaves.

*Peumus boldus* Mol., (Monimiaceae) is a Chilean medicinal tree used for gastrointestinal and liver diseases [1,2]. In Chile, this tree also is called Boldo, Peta, Voldu or Boldu and botanically was described for the first time by Molina in 1782. The phytochemical profiling of Boldo usually is based on its aporphine alkaloids and phenolic compounds, whose concentration varies depending on the analyzed part of the tree [3,4,5,6]. For example, the concentration of boldine is higher in the bark than in the leaves. Moreover, certain classes of alkaloids are concentrated in other parts such as wood, fruits or roots and it is not possible to detect them in the leaves [7]. So, the analysis of botanical parts of Boldo tree allowed the identification of alkaloids such as boldine, isoboldine, coclaurine, *N*-methylcoclaurine, (−)-pronuciferine, (+)-reticuline, laurotetanin, *N*-methyl-laurotetanin, sinoacutin, isocorydine, isocorydine *N*-oxide, nor-isocorydine and laurolitsin (norboldine) (Figure 1). However, pharmacopoeias (European and British) and EMA reports have established a minimum limit of 1.0 per cent of total alkaloids in Boldo leaves expressed as boldine (dry weight basis) [8,9]. Among flavonoids, rhamnetin 3-*O*-arabinoside 3′-*O*-rhamnopyranoside (peumoside), isorhamnetin 3-*O*-glucoside 7-*O*-rhamnoside (boldoside), kaempferol-3-glucoside-7-rhamnoside, isorhamnetin di-glucosyl-di-rhamnoside and other quercetin and kaempferol glycosides have been reported [6,10]. Due to its higher concentration and better extraction yields in herbal infusions, some authors thought that flavonoids are responsible for the antioxidant and chemopreventive effects of Boldo [6,10,11]. Moreover, Boldo leaves also have higher amounts of gallic acid, tannins and catechin-derived procyanidins rather than boldine and other alkaloids. However, so far, no pre-clinical or clinical evidences have been generated to confirm such statement. The mild choleretic activity of isolated boldine (50 mg kg^−1^day^−1^ dose) has been ascribed to osmotic effect and an up-regulation of Bsep transport mediated by farnesoid X receptor activation [12]. Also, a recent work suggest that boldine (50 mg kg^−1^day^−1^, oral gavage) have renoprotective activity in hypertensive 2K1C rats reducing plasmatic levels of thiobarbituric acid reactive substances (TBARS) and lowering the increase in angiotensine converting enzyme type I (ACE-1). This effect leads to a decreasing of several downstream mediators such as transforming growth factor beta (TGF-β) [13]. By another hand, it has been reported that boldine has in vitro antioxidant and trypanocidal activities [6,14,15,16]. Another study show that the most abundant alkaloid found in Boldo leaves was isocorydine, which currently is under evaluation for its anticancer properties [17]. An in vitro study conducted by our group found that catechin-based proanthocyanidins (dose of 15.9 µg gallic acid equivalents mL^−1^) are potent inhibitors against *Helicobacter pylori* urease and bacterial adherence (dose of 2.0 mg gallic acid equivalents mL^−1^) to human gastric adenocarcinoma cells (AGS), whereas boldine and ascaridol were inactive [18]. In the last five years, new solvents have been introduced as an alternative to conventional organic solvents. Among them, Deep Eutectic Solvents (DES) are generally less toxic and include not only synthetic compounds but also several organic compounds of natural origin such as choline chloride or malic acid [19,20,21,22]. These components could be simple sugars (sucrose, glucose, fructose), organic acids (malic, citric, lactic acid), amino acids (proline, glycine) or quaternary ammonium compounds (choline or betaine), which normally are concentrated inside or around vesicles in vegetal cells [23]. So, it is believed that DES formed inside the plant cell play a central role in improving the solubility of water insoluble molecules such as lignin, terpenes and a wide type of aglycons. Indeed, when some of these metabolites were combined in certain proportions, viscous liquids are generated, which are called Natural Deep Eutectic Solvents (NADES). In general, the components of NADES are characterized by the presence of various functional groups such as hydroxyl, carboxyl or amino groups [23,24]. NADES are typically formed between a hydrogen bond donor compound (HBD) and a hydrogen bond acceptor compound (HBA). These groups can form an intermolecular hydrogen bond, leading to highly structured viscous liquids, which explains their specific physical properties and their different solubilizing behavior compared to conventional solvents such as water and alcohols. Therefore, these liquids can form hydrogen bonds with phenolic compounds increasing significantly their solubility in the NADES network structure. They have been shown many advantages over the conventional solvents currently used for extraction. For instance, they have vapor pressure near zero, adjustable viscosity and high solubilization ability, which in some cases can exceed 12,000 times that of water [23,24]. From the environmental and economic point of view, they also present major advantages with respect to their biodegradability, sustainability, low cost and simple preparation. All these properties suggest their great potential as extraction media for natural products and their possible applications for foods, pharmaceuticals and cosmetics [25]. The use of NADES in the extraction of alkaloids is still very limited and there are only a few reports of its use published in the last two years. One of the most recent works deals with the extraction of alkaloids derived from morphinane, protoberberine, bisbenzylisoquinoline, indole and quinolizidine alkaloids using 75 different DES [26]. Authors found that DES based on Choline-lactic acid were superior for the extraction of alkaloids derived from morphinane, protoberberine, indole, and quinolizidine alkaloids. It is worth noting that one of the parameters that most influenced the extraction with DES was the water content used, which was optimized at close to 46%. In another recent work, different NADES were used for the extraction of alkaloids from Amarillydaceae [27]. These authors reported that NADES with the best efficiency to extract lycorine, crinine and crinamine were those derived from Choline Chloride: fructose and H_2_O (35%). The optimization of such solvent showed that the best conditions were: a temperature of 45 °C, extraction time of 51 min and a water content of 21%. Moreover, the same group evaluated the cytotoxicity of extracts made with NADES and surfactants for the same alkaloids, finding that the solvent can significantly influence the biological activity of the extract [28]. Considering the above mentioned variables, in this work we assess for the first time the extraction of the alkaloids and polyphenols from Boldo leaves using different NADES. These new green solvents not only improve the extraction of bioactive compounds but also allow to obtain extracts with different phytochemical profiles. Additionally, in the present work, we evaluated the presence of the main alkaloids and phenolic compounds present in *P. boldus* leaves extracts by using HPLC-DAD-IT-MS/MS and HPLC-QTOF-MS/MS.

## 2. Results and Discussion

### 2.1. Phytochemical Profiling of Peumus boldus Methanol Extract

**Figure 2:**
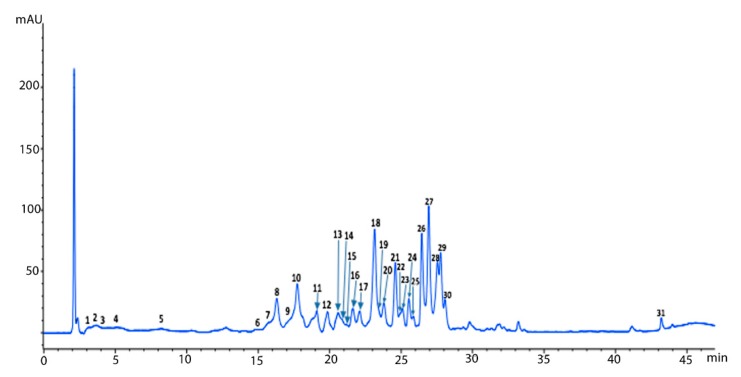
Representative HPLC-PDA trace of *Peumus boldus* leaf extract obtained with methanol. The trace correspond to the signals recorded at 304 nm. Numbers refers to the compounds listed in Table 1 and Table 2. HPLC separation was performed in reverse phase under gradient.

Figure 1 shows the structure of the two main types of alkaloids present in *P. boldus*. In Figure 2 an illustrative chromatogram is shown for the methanol extract (control solvent) of *P. boldus* registered at 304 nm. Peaks were numbered according to its elution order from 1 to 31. As summarized in Table 1 and Table 2 as well as Figures S1–S4, identification was based on UV spectra obtained by HPLC-PDA, comparison of the retention times with available standards, accurate masses (HPLC-QTOF-MS/MS) and MS/MS spectra (HPLC-IT-MS/MS). Therefore, these chromatographic analyses allow the identification of nine alkaloids. Among these compounds, six were identified as aporphines: laurolitsine, isoboldine, boldine, isocorydine, laurotetanine and *N*-methyllaurotetanine; and three were identified as benzylisoquinoline derivatives: coclaurine, *N*-methylcoclaurine and reticuline. Additionally, 22 phenolic compounds were identified by HPLC-IT-MS/MS in *Boldo* leaf extracts (Table S1). Most of them had been previously reported by Simirgiotis and coworkers [6], who identified 52 phenolic compounds in male and female *Boldo* trees. As expected, since this author used aqueous extraction, a greater presence of proanthocyanidins oligomers (19 trimers + tetramers) is plausible. Also, a greater number of tri and tetra-glycosides of quercetin, isorhamnetin and kaempferol was observed.

#### 2.1.1. Identification of *P. boldus* Phenolic Compounds

In our study, compounds corresponding to the chromatographic peaks 1, 2 and 5 show molecular ions [M + H]^+^ at *m*/*z* 579 and the characteristic MS/MS ion fragment at 291, suggesting that these compounds are procyanidin dimers of catechin or epicatechin (compounds corresponding to the chromatographic peaks 3 and 4, *m*/*z* 290.4 and 291.1). In a previous work, after phloroglucinolysis, we demonstrate that the structure of such compounds corresponds to catechin-derived procyanidins [18]. Compounds corresponding to the chromatographic peaks 11, 16, 17, 18, 23, 25, 26 were identified as luteolin derivatives. Compound corresponding to chromatographic peak 11 was identified as luteolin-pentosyl-glucosyl-rhamnose with molecular ion [M + H]^+^ at *m*/*z* 727.2. MS/MS data confirm a loss of 132 amu (dehydrated pentose) and a subsequent loss of 162 amu (hexose) and 146 amu (rhamnose). Peak 16 was identified as luteolin 3-*O*-rutinoside with molecular ion [M + H]^+^ at *m*/*z* 595.1. MS/MS data confirm a loss of 162 amu (hexose) and 146 amu (rhamnose). Peak 17 was identified as luteolin dipentosyl rhamnoside with molecular ion [M + H]^+^ at *m*/*z* 697.2. MS/MS data confirm a sequential loss of two 132 amu (dehydrated pentose) and 146 amu (rhamnose). Compound corresponding to peak 18 was identified as luteolin 7-*O*-rutinoside with molecular ion [M + H]^+^ at *m*/*z* 595.1. MS/MS data confirmed a loss of 162 amu (hexose) and 146 amu (rhamnose). Peak 23 was identified as luteolin with molecular ion [M + H]^+^at *m*/*z* 286.2. Peak 25 was identified as luteolin glycoside with molecular ion [M + H]^+^at *m*/*z* 595.1. However, MS/MS data do not allow to confirm its identity. Compound 26 was identified as luteolin dirhamnoside with molecular ion [M + H]^+^at *m*/*z* 579.1. MS/MS data confirmed a sequential loss of two 146 amu (rhamnose). Compound corresponding to peak 12 was identified as hesperidin-7-*O*-rhamnoglucoside with molecular ion [M + H]^+^at *m*/*z* 611.1. MS/MS data confirm a loss of 162 amu (hexose) and a subsequent loss of 146 amu (rhamnose). Peak 14 is a quercetin derivative with molecular ion [M + H]^+^at *m*/*z* at 581.1. MS/MS data of this compound suggested a loss of 132 amu corresponding to dehydrated pentose and a subsequent loss of 146 amu (deoxyhexose), giving the diagnostic fragment of quercetin at *m*/*z* 303 in positive mode. This compound was reported previously as quercetin pentosyl-rhamnose [6]. Peaks 13, 19, 24 and 29 were tentatively identified as myricetin derivatives. Peak 13 was identified as myricetin-rhamnosyl-glucosyl-pentoside with molecular ion [M + H]^+^ at *m*/*z* 757.2. MS/MS data confirm the sequential loss of 132 amu (dehydrated pentose), 162 amu (hexose) and 146 amu (rhamnose). Peak 19 was identified as myricetin-rhamnosyl-hexose with molecular ion [M + H]^+^ at *m*/*z* 625.2. MS/MS data confirm the sequential loss of 162 amu (hexose), and 146 amu (rhamnose). Peak 20 was identified as myricetin pentosyl-hexosyl-rhamnoside with molecular ion [M + H]^+^ at *m*/*z* 727.1. MS/MS data confirm the sequential loss of 132 amu (dehydrated pentose), and 162 amu (hexose) and 146 amu (rhamnose). Compound corresponding to peak 24 was identified as myricetin-rhamnosyl-pentoside with molecular ion [M + H]^+^ at *m*/*z* 595.2. MS/MS data confirm the sequential loss of 132 amu (dehydrated pentose), and 146 amu (rhamnose). Peak 29 was identified as myricetin-dirhamnoside with molecular ion [M + H]^+^ at *m*/*z* 609.1. The MS/MS data confirm the sequential loss of two 146 amu (rhamnose). Peak 14 was identified as quercetin pentosyl-rhamnoside with molecular ion [M + H]^+^ at *m*/*z* 581.1. MS/MS data confirm the sequential loss of 132 amu (dehydrated pentose), and 146 amu (rhamnose). Peak 22 was identified as isorhamnetin rhamnosyl-glucosyl-rhamnoside with molecular ion [M + H]^+^ at *m*/*z* 771.2. MS/MS data confirm the sequential loss of 146 amu (rhamnose), 162 amu (hexose) and 146 amu (rhamnose). Peaks 30 and 31 are kaemferol glycosides. Peak 30 was identified as kaempferol-3-*O*-glucosyl-rhamnosyl-rhamnose with molecular ion [M + H]^+^ at *m*/*z* 741.2. MS/MS data confirm the sequential loss of 132 amu (dehydrated pentose) and two 146 amu (rhamnose). Peak 31 was identified as kaempferol-3-*O*-coumaroyl-rhamnoside with molecular ion [M + H]^+^ at *m*/*z* 595.1. MS/MS data confirm the loss of 308 amu (coumaroyl glucoside moiety).

#### 2.1.2. Identification of *P. boldus* Alkaloids.

As is shown in Table 1 and Table 2, for compounds corresponding to the peaks 6 and 7 in positive ionization mode, the molecular formulas of ions at *m*/*z* 285.13653 and 299.15228 were predicted as C_17_H_19_NO_3_ and C_18_H_21_NO_3_. In HPLC-PDA-IT-MS/MS analysis, peaks 6 and 7 shown precursor ions at 286.1 [M + H]^+^ and *m*/*z* 300.1 [M + H]^+^ yielded the MS/MS fragments ions at *m*/*z* 269, which correspond to the loss of NH_3_ and CH_3_NH_2_, respectively. These data agrees with the fragmentation patterns of coclaurine and *N*-methylcoclaurine [29,30,31]. In addition to this fragmentation pattern, these compounds suffer neutral loss of CH_3_OH, which explain the fragment ion at *m*/*z* 237. Subsequently this last fragment gave the ion at *m*/*z* 209 corresponding to –CO loss. Fragment ion at *m*/*z* 175 could be explained by the cleavage of the double bond present in the mother fragment ion at *m*/*z* 269. Finally, β-cleavage of the fragment ion at *m*/*z* 269 explain the origin of the ion fragment at *m*/*z* 137 (see scheme in Figure S4). On the other hand, for compound corresponding to peak 8 in positive ionization mode, the molecular formula of ion at *m*/*z* 313.13653 was predicted as C_18_H_19_NO_4_. In HPLC-PDA-IT-MS/MS analysis, peak 8 show a precursor ion at *m*/*z* 314 [M + H]^+^ and in MS/MS gave a fragment ion at *m*/*z* 297 corresponding to the loss of 17 Da [MH + NH_3_]^+^ and a main fragment ion at *m*/*z* 265 generated by the sequential loss of two methyl radicals. Subsequently this last fragment gave the ion at *m*/*z* 237 corresponding to –CO loss. These data suggests that peak 8 is laurolitsine [32]. For compounds corresponding to peaks 9 and 10 in positive ionization mode, the molecular formulas of ions at *m*/*z* 327.14718 and 327.14716 were predicted as C_19_H_21_NO_4_ and C_19_H_21_NO_4_. In HPLC-PDA-IT-MS/MS analysis both compounds share the same molecular ion at *m*/*z* 328.1 [M + H]^+^ and in MS/MS gave the same fragments ions at *m*/*z* 297 [MH + H-31]^+^ corresponding to the loss of 31 Da from CH_3_NH_2_ and also a main fragment ion at *m*/*z* 265 [M + H-31-32]^+^ and *m*/*z* 237 [M + H-31-32-28]^+^, generated by the sequential loss of two methyl radicals and 28 Da from –CO loss, respectively. These data and the comparison of elution order for standards in C-18 columns suggest that the identity of peaks 9 and 10 could unambiguously be assigned to isoboldine and boldine [29,31,33,34]. For compound corresponding to peak 15 in positive ionization mode, the molecular formula of ion at *m*/*z* 329.16323 was predicted as C_19_H_23_NO_4_. In HPLC-PDA-IT-MS/MS analysis, peak 15 showed a molecular ion at *m*/*z* 330 [M + H]^+^ and in MS/MS a prominent product ion at *m*/*z* 192 [M + H-138]^+^, which is consistent with the loss of C ring with methoxyl and hydroxyl groups previous to a putative loss of 31 Da from CH_3_NH_2_ (very low abundance of fragment at *m*/*z* 299). In addition, fragment ion at *m*/*z* 192 is a diagnostic ion used to confirm the presence of a methoxyl and hydroxyl groups at the A ring in benzylisoquinoline alkaloids [31,35,36]. According with MS data, peak 15 is reticuline. For compound corresponding to peak 21 in positive ionization mode, the molecular formula of ion at *m*/*z* 341.16282 was predicted as C_20_H_23_NO_4_. In HPLC-PDA-IT-MS/MS analysis, peak 21 showed a molecular ion at *m*/*z* 342.1 [M + H]^+^, ions at *m*/*z* 311 and 296 in MS/MS product and a prominent fragment ion at *m*/*z* 279 caused by the sequential loss of CH_3_NH_2_ and a methoxyl radical. Fragments ions at *m*/*z* 264 and *m*/*z* 248.1 correspond to the consecutive loss of two methyl groups. According to these data, the identity of peak 21 is assigned to isocorydine [36]. For the compound corresponding to peak 27 in positive ionization mode, the molecular formula of ion at *m*/*z* 327.14735 was predicted as C_19_H_21_NO_4_. In HPLC-PDA-IT-MS/MS analysis, peak 27 yielded a molecular ion [M + H]^+^ at *m*/*z* 328 and MS/MS ion at *m*/*z* 311 corresponding to the loss of 17 Da [MH + NH_3_]^+^. The ion fragment at *m*/*z* 279 is generated by a sequential loss of two methyl radicals, whereas the fragment ion at *m*/*z* 248 could be generated by the loss of a methoxyl group. These data and the elution time suggest that this peaks is laurotetanine. Similarly, for peak 28 in positive ionization mode, the molecular formula of ion at *m*/*z* 341.16313 was predicted as C_20_H_23_NO_4_. In HPLC-PDA-IT-MS/MS analysis, peak 28 shown a molecular ion [M + H]^+^ at *m*/*z* 342. In MS/MS, this compound afforded the product ion at *m*/*z* 311 [MH+H-31]^+^ corresponding to the loss of 31 Da from CH_3_NH_2_. The observed fragments ions at *m*/*z* 296, 280 and 265 are coherent with the consecutive loss of three methyl groups. These data and the elution time suggest that this peak is *N*-methyl-laurotetanine (rogersine) [29,32]. Proposed fragmentation of aporphine alkaloids of *P. boldus* can be observed in the scheme presented in Figure S5.

### 2.2. Extractability of Boldine from Peumus boldus Leaves with Diverse NADES

**Figure 3:**
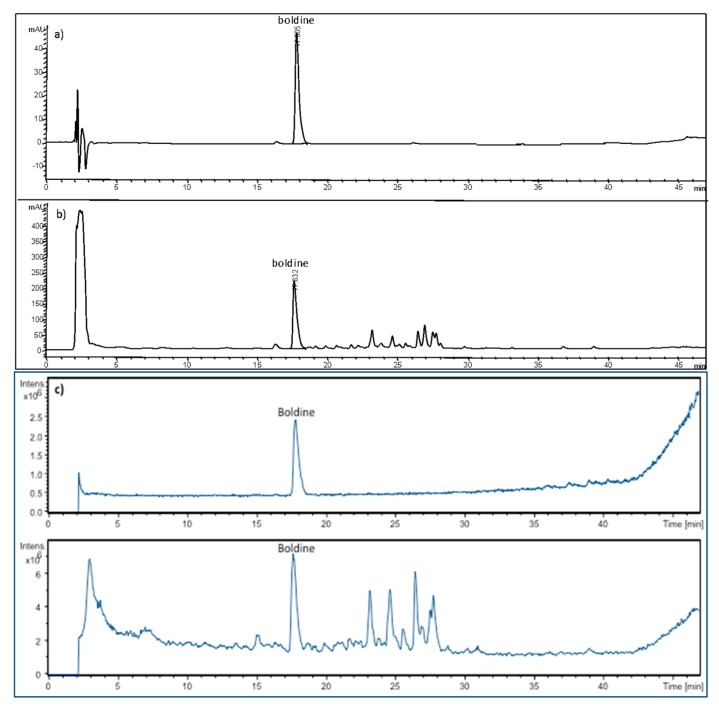
Illustrative HPLC-PDA chromatograms of boldine standard (**a**) and *Peumus boldus* leaves extracted with NADES6 (**b**). HPLC-IT/MS chromatograms of boldine standard and *Peumus boldus* leaves extracted with NADES6 (**c**). HPLC separation was performed in reverse phase under gradient. The trace correspond to the signals recorded at 304 nm.

Once identified the characteristic alkaloids in *Boldo* leaves, their extraction with seven selected NADES was evaluated (Table 3) [25,26,27,28,37,38,39,40,41,42,43]. The efficiency of these NADES was compared with methanol and water. If the literature related to Boldo extraction is carefully reviewed, it can be seen that both methanol and ethanol are conventionally used solvents and therefore we selected methanol as the control solvent (Table 4). For example, Rogalisnki and coworkers [44], evaluated the performance of boldine extraction using hot pressurized water and supercritical CO_2_ compared with methanol extraction. Also in the work of Fuentes-Barros [7], the extraction of *P. boldus* alkaloids for HPLC analysis was performed with methanol. As in shown in Figure 3b, in the chromatogram obtained with NADES6, boldine appeared at around t_R_ = 18 min. For the quantitative analysis of boldine in the different NADES extracts, we used a UV signal (304 nm) from HPLC-DAD-IT/MS (Figure 3a,b).

**Figure 4:**
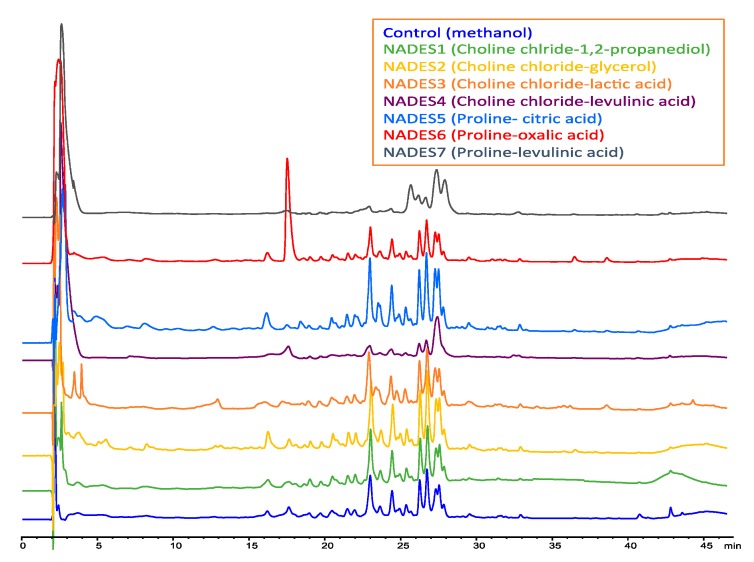
HPLC-PDA chromatograms of the alkaloids from *Peumus boldus* leaves extracted with different NADES solvents. HPLC separation was performed in reverse phase under gradient. The trace correspond to the signals recorded at 304 nm.

**Figure 5:**
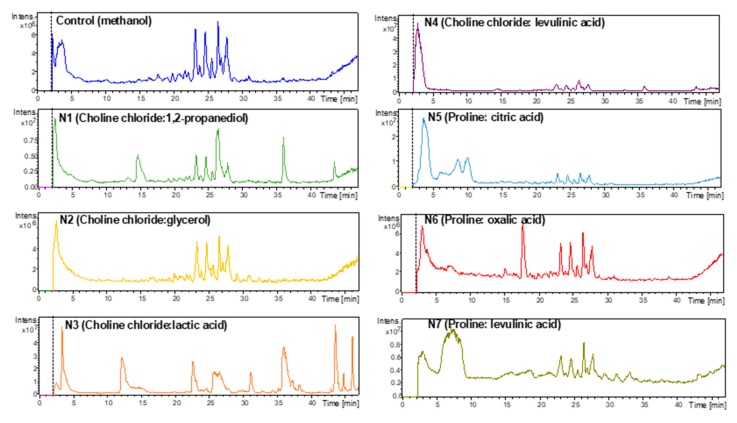
Representative HPLC-ESI-IT-MS chromatograms of the alkaloids from *Peumus boldus* leaves extracted with different NADES solvents. Control solvent (methanol) correspond to the chromatogram depicted in the first frame traced in blue color.

So, in Figure 4 and Figure 5 are illustrated the chromatographic profiles obtained with NADES 1-7 compared with the methanol extract. A prominent peak of boldine (t_R_ = 17.8 min) can be observed for NADES6 in the UV trace (304 nm) and IT-MS detection ([M + H]^+^ = 328.1).

**Figure 6:**
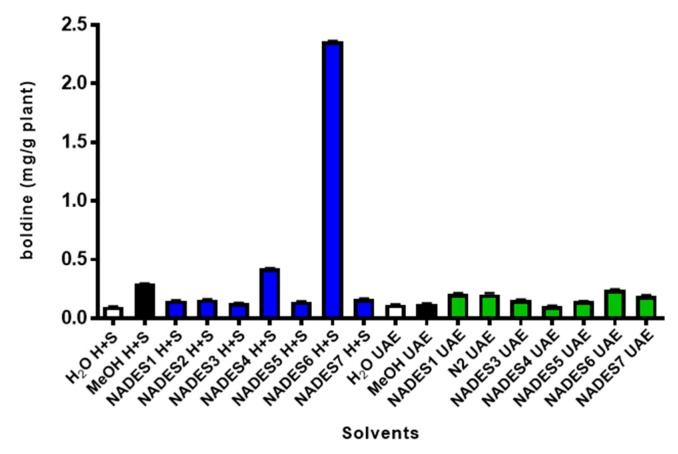
Effect of different NADESs on the extraction of boldine from *Peumus boldus* leaves with using heat + stirring (H+S, blue bars) and ultrasonic assisted extraction (UAE, green bars). In all extraction performed with NADES 1-7, 20% water was added to reduce viscosity. White and black bars corresponds to extraction performed with water and methanol, respectively.

Figure 6 presents the quantitative analysis of boldine, where it is clear that methanol is two-times more efficient than NADES1, NADES2, NADES3, NADES5 and NADES7 (0.1533 mg, 0.1607 mg, 0.1291 mg, 0.1473 mg and 0.1650 mg g^−1^ dry plant). NADES1 and NADES2 are alcohol-based solvents with polarity quite similar to ethanol [26,27,28]. Interestingly, we found two NADES that enable better extraction of boldine from *Boldo* leaves. These solvents were NADES4 (choline chloride-levulinic acid, 1:1) and NADES6 (proline–oxalic acid, 1:1) with 0.4270 mg and 2.3615 mg of boldine per gram of plant, respectively. From these results, it is remarkable that boldine extraction with NADES6 is eight-times more efficient than methanol. Moreover, boldine extraction yields varied greatly depending on the type of HBD used for NADES preparation. For instance, the alcohol-based NADES exhibited poor extraction capacity, reaching only a 54% alkaloids in comparison with methanol. On the other hand, the extraction efficiencies of carboxylic acid-based such as NADES4 and NADES6 were significantly higher than other NADESs, as well as methanol.

These results are in agreement with the results published by Duan and coworkers [37], for other types of alkaloids such as jatrorrhizine hydrochloride, palmatine hydrochloride and berberine hydrochloride from herb Berberidis Radix. The differences observed between NADESs and MeOH can be due to the lack of extractability of partially ionized compounds by MeOH, where electrostatic interactions could significantly contribute to their extraction [38]. Then, when this result is compared with other extraction methods [7,10,45,46,47,48,49], it is observed that the extraction performance for boldine is still more efficient than the other extraction procedures based on conventional solvents (Table 4). This difference can be explained by the variations in the substrate, the extraction procedure or method of analysis. With respect to the extraction process, the difference may be due to the limited selectivity of the adopted method and the resulting contamination of the alkaloid fraction and -at least in part- to the improvements in the solubility of the alkaloids. The latter can be explained by the increase in the solvation of non-polar organic solvents for alkaloids (naturally present as salts in *Boldo* leaves) after pH adjustment of aqueous alcohol solutions in the pretreatment steps. For instance, in acidic conditions, boldine is protonated and its water solubility is significantly better than in the neutral solvent [26,37].

### 2.3. Extraction Yields of Total Polyphenols from Peumus boldus Leaves

**Figure 7:**
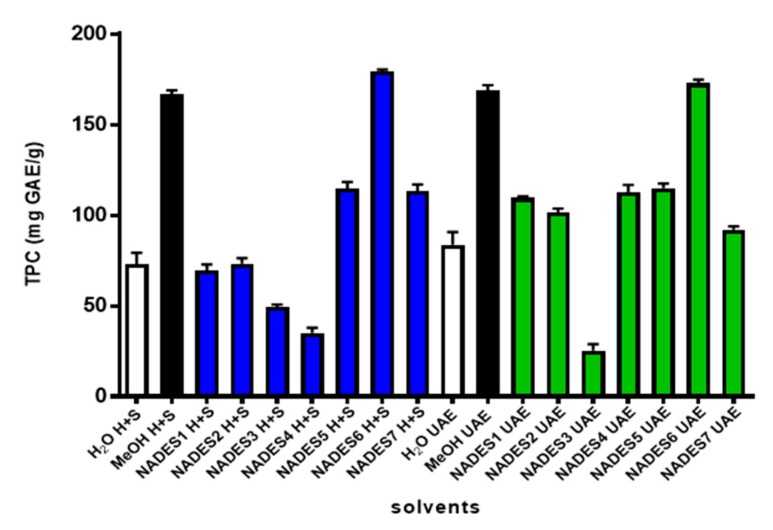
Effect of different NADESs on the extraction of polyphenols from *Peumus boldus* leaves using heat + stirring (H+S, blue bars) and ultrasonic assisted extraction (UAE, green bars). In all extraction performed with NADES 1λ7, 20% water was added to reduce viscosity. White and black bars corresponds to extraction performed with water and methanol, respectively.

In Figure 7, it is observed the results obtained for total content polyphenols (TPC) are expressed as gallic acid equivalents (GAE). The best yield for the extraction of total polyphenols was again obtained with the NADES6 (L-proline: oxalic acid). Interestingly, no significant difference was observed in the results of TPC between heating + stirring extraction (179.442 ± 3.79 mg g^−1^ GAE dw) and UAE extraction (172.659 ± 2.55 mg g^−1^ GAE dw). Moreover, the TPC in extracts obtained with control solvent (methanol) does not show significant differences with NADES6 (one-way ANOVA, *p* < 0.05), suggesting that NADES6 could be used to replace methanol. On the other hand, our results showed that H_2_O, NADESs 1-4 and 7 were less suitable for the extraction of polyphenols. Since high viscosity of NADES is one of the main drawbacks for its use, it is worth mentioning that in all the extractions performed in the present study the viscosity was reduced by adding a maximum of 20% of water (Table 3). It has been reported that this strategy does not alter the supramolecular NADES network; on the contrary, it has a dramatic effect by increasing the mass transfer and mobility of the molecules [41,42]. Therefore, the addition of water combined with temperature help to reduce the strong intermolecular interactions ruled by the H bond network in eutectic solvents [43,50].

NADESs prepared with levulinic acid-choline chloride- or 1, 4 butanediol also give good results and could be used too in combination with more exhaustive methods such as ultrasound and microwave-assisted extraction [49,50,51]. In the case of TPC, NADES6 (H+S and UAE) does not shown significant differences when compared with control solvent (methanol) and could be used as a greener replacement for this solvent (Figure 7). It should be noted that viscosity and negligible volatility of NADES are two properties that constitute a disadvantage compared to traditional solvents (methanol or ethanol). This disadvantages has led to profusely search new eutectic solvents with low viscosity. On the other hand, the volatility of traditional solvents allows their distillation, but in turn it is an environmental problem since they can cause air pollution and cause damage to human health. While it is true, solvents such as water, ethanol or methanol are cheaper and widely used solvents, they have some additional drawbacks. In particular, although they can be as efficient as a NADES, these alcohols have the serious disadvantage of being flammable and in cases where large-scale extraction processes must be scaled, they are considered to be dangerous. Nevertheless, it is clear that there are several points of debate regarding the use of NADES which require attention in the future. For instance, it is necessary to know more about the toxicity and permanence of NADES residues in the environment (degradation) and how these residues could affect living organisms. Furthermore, the use of NADES as bio-compatible solvents require to know if they affect the bioactivity of the bioactive products. The removal or recovery of NADES are points frequently addressed in recent publications. However, several strategies have been proposed. For instance, Liu and coworkers [52] reported the application of counter-current separation (CCS) to recover secondary metabolites from NADES. They also propose use CCS to recycle NADES because it remain intact after CCS and can be extruded from the column of high speed counter current chromatography (HSCCC) apparatus. Liquid-Liquid extraction also has been recently proposed by Smink et al. [53]. Other methods such as ultrafiltration, precipitation, solid phase extraction and electro-dialysis have been reported for NADES [54,55,56,57,58].

## 3. Materials and Methods

### 3.1. Chemicals and Reagents

Choline chloride (>98.0%), l-(+)-Lactic acid (>98.0%), glycerol (>99.5%), 1,2-propanediol (>99%) and 2,9-Dihydroxy-1,10-dimethoxyaporphine (boldine) analytical standard (purity ≥ 98%), were purchased from Sigma-Aldrich (Steinheim, Germany). Citric acid (>98%), levulinic acid (>98%), l-Proline (>99%), oxalic acid (>99%), sodium carbonate (>99.9%), gallic acid (>98.0%), and Folin–Ciocalteu’s phenol reagent for analysis-grade were obtained from Merck (Darmstadt, Germany). HPLC-grade acetonitrile, methanol, formic acid, ammonium formate were obtained from Merck (Darmstadt, Germany). (Sigma Aldrich, Saint Louis) was used as references for identification. Ultrapure water was produced by a Milli-Q apparatus (Millipore, Bedford, MA, USA).

### 3.2. Preparation of NADES

The preparation of all NADES tested was based on previously reported procedures [24,25,26,37,38]. Briefly, choline chloride and l-proline (hydrogen bond donor—HBD) was mixed with lactic acid, 1,2-propanediol, glycerol, levulinic acid, citric acid or oxalic acid (hydrogen bond acceptors—HBA) at predetermined molar ratios. Mixtures were mildly heated under stirring, until a perfectly transparent liquid was formed. NADES were kept in sealed glass vials in the dark, at ambient temperature. The list of the NADES used in this study, along with details regarding their preparation and references, are presented in Table 3.

### 3.3. Plant Material and Extraction

The plant material (leaves of *Peumus boldus*) was collected at the University of Concepción in June 2017 and authenticated in the Herbarium of the Department of Botany at the University of Concepción, Chile. The Voucher specimen was kept under code CONC N° 187541. After collection, the plant samples were air dried for 14 days at room temperature in the dark, and then ground to a fine powder using a Blender (Waring, McConnellsburg, PA, USA). This material was used for all further procedures. All NADES were used as 80% (*v*/*v*) aqueous solutions in order to reduce viscosity. Extractions were carried out according to a previously described methodology [39,40]. In brief, plant material (0.1 g) was placed in a 50 mL conical centrifuge tubes and 10 mL of NADES solvent was added. With the aim to compare the extraction yield of alkaloids from *P. boldus*, methanol and water were used as control solvents under the same conditions set up for all NADES. The suspension was vortexed vigorously for 30 s until a homogeneous thick mixture was obtained. Then, homogeneous samples were extracted through Heating and stirring extraction in a Syncore Polyvap R24 (Büchi, Flawil, Switzerland), under the following conditions: 60 °C for 50 min at 340 rpm. In addition, Ultrasound-assisted extraction (UAE) was performed using an Ultrasonic homogenizer bar JY92-IIDN (XinZhi Institute, NingBo, China) at room temperature for 20 min with a sonication power of 140 W and frequency of 37 kHz. After extraction, samples were clarified by centrifugation (Eppendorf 5804 R, Long Island, New York, NY, USA) at 8000 rpm for 10 min. The supernatants were filtered through a Millipore 0.45 μm cellulose acetate membrane filter and two-fold diluted with mobile phase prior to HPLC analysis. The extraction procedure describes above was performed in triplicates. For total polyphenol content determination, samples were 10-fold diluted with distilled water.

### 3.4. Analysis of Extracts

#### 3.4.1. Total Polyphenol Content

The extracts were re-dissolved in water, and total phenolic content (TPC) was determined by using the Folin–Ciocalteu method with slight modifications [59]. In brief, 20 μL of properly diluted samples were mixed with 780 μL of distilled water and 50 μL of Folin-Ciocalteu reagent in 1.5 mL conical tubes. After 1 min, 150 μL of 7.5% sodium carbonate solution were added and mixed. Samples were leave in the dark at room temperature for 1 h. Aliquots of 200 μL were charged in 96 well microplates and the absorbance was measured at λ 750 nm using an EPOC microplate reader (Biotek). The analysis were performed in triplicate and normalized against negative controls (distilled water or diluted NADES) according to the Table 1. TPC was expressed as milligrams of gallic acid equivalents per gram of extract (mg g^−1^ GAE of extract) based on a standard curve of gallic acid (50–400 mg L^−1^; y = 0.013x + 0.0073; R^2^ = 0.9991).

#### 3.4.2. Qualitative and Quantitative HPLC-PDA-IT-MS/MS Analysis

The samples of *P. boldus* were analyzed by HPLC-PDA-IT-MS/MS in an Agilent 1100 Series system (Agilent, Waldbronn, Germany) equipped with an automatic degasser, a quaternary pump, an auto-sampler and a photodiode array detector (G1315B) and LC/MSD Trap VL (G2445C VL) ESI-MS^n^ system, and it was coupled to an Agilent Chem Station (version B.01.03) data-processing station. The stationary phase employed was a Zorbax Eclipse XDB-C18 Narrow-Bore (150 mm × 2.1 mm; 3.5 μm particle size) column, while the mobile phase consisted of solvent A (water/formic acid/acetonitrile, 87:10:3, *v*/*v*/*v*) and solvent B (acetonitrile/water/formic acid, 50:40:10, *v*/*v*/*v*). The elution profile was (time, % of solvent B): 0 min, 3%; 10 min, 15%; 35 min, 40% B; 39–41 min, 100% B, and 47 min, 3% B [60]. The flow rate was 0.190 mL min^−1^ and the column temperature was set at 40 °C while the injection volume was 20 μL. The mass spectrometer was run in the positive ion mode with the following parameters: the capillary voltage was set at 3500 V, drying gas flow N_2_, 8 mL min^−1^; drying temperature, 325 °C; nebulizer, 50 psi; and scan range, 100–1200 *m*/*z*. The collision energy (CE) increased linearly in the range of 30–45 eV depending on the *m*/*z* range (100–1200). The range of detection wavelength were 200–600 nm. However, for alkaloids detection and boldine quantification, 304 nm was selected. *Boldo* extracts (10 mg) were dissolved in methanol (1 mL), diluted with mobile phase and filtered with a 0.45 μm syringe filter of polytetrafluoroethylene (13 mm) (Millex). The results were expressed as milligrams per gram of extract (mg g^−1^ extract). The linearity of the method was assessed from the correlation coefficients (R^2^) of three set of calibration curves obtained for seven levels of boldine concentrations ranging from 0.0469 mg L^−1^ to 15.00 mg L^−1^ (y = 352.33x − 75.904; R^2^ = 0.9971). Each point was injected three times. Limit of detection (LOD) and Limit of quantification (LOQ) were estimated at signal to noise (S/N) ratios of 3:1 and 10:1, respectively [61]. With this procedure, LOD and LOQ values were 0.003 mg L^−1^ and LOQ = 0.023 mg L^−1^, respectively.

#### 3.4.3. Q-ToF High-Resolution Mass Spectrometry Measurements

The analytical system used consisted of a 1260 Infinity high performance liquid chromatography system coupled to a diode array detector (DAD) and a 6545 quadrupole-time of flight (Q-TOF) mass spectrometer detector (Agilent, Waldbronn, Germany). The control software was Mass Hunter Workstation version B.06.11 (Agilent, Santa Clara, CA, USA). The Q-TOF used a Dual Jet Stream Electrospray Ionization (Dual AJS-ESI) source operated in the positive ionization mode and the following parameters were set: capillary voltage, 3500 V; fragmentor, 200; gas temperature, 350 °C; drying gas, 8 L min^−1^; nebulizer, 40 psig; sheath gas temperature, 400 °C; sheath gas flow, 10 L min^−1^; acquisition range, 100–1000 *m*/*z*; and CID, with a linear range of 30–45. Samples were analyzed after injection (10 μL) on a Zorbax Eclipse Plus C18 Rapid Resolution HD column (2.1 mm × 50 mm, 1.8 μm) protected with a 5 mm guard column of the same material thermostated at 40 °C and his flow rate was 0.3 mL min^−1^. The solvent system was 1mM of ammonium formate + 0.1% formic acid in water (solvent A) and 1 mM of ammonium formate + 0.1% formic acid in methanol (solvent B). The elution gradient was (time, % of solvent B): 0 min, 7%; 10 min, 20%; 40 min, 75%; 46.5 min, 95%; 56 min, 7%; and a post time of 8 min. Compounds were identified using the algorithm “Find by Formula” that evaluated the mass accuracy together with the isotopic relative abundance and isotopic separation. All the compounds were identified by the QTOF-MS and the MS/MS spectra acquired with the IT-MS and their absorption spectra in UV-visible region, as well as considering the data provided by literature [6,7,29,30,31,32,33,34,35,36].

### 3.5. Statistical Analysis

Statistical comparison was performed using GraphPad Prism 5. (GraphPad Software, San Diego, CA, USA). Variables were expressed as mean and standard deviation (SD). The comparisons between the means in each assay were performed by one-way analysis of variance (ANOVA) at a 95% confidence level. Tukey’s multiple comparison post-hoc test was applied to determine the differences amongst extraction yields. Data points plotted in Figure 5 and Figure 6 represent the means of at least three independent experiments, each conducted in triplicate.

## 4. Conclusions

In this work, advanced analytical methods have been used to carry out a thorough characterization of a *P. boldus* extracts. In this report, the identity of main *P. boldus* alkaloids and phenolics compounds was confirmed by HPLC coupled to DAD-IT-MS/MS and Q-ToF HRMS. Finally, from our results, it can be concluded that NADESs are a potential green alternative to conventionally used organic solvents as extraction media to improve the extraction of alkaloids and phenolic compounds. Among the NADESs tested in our study, proline-oxalic acid (1:1) with 20% water was the most promising solvent, attaining higher extraction yields of boldine and TPC from *P. boldus* leaves. Overall, an adequate fine-tuning of HBD/HBA components in a NADES is a powerful strategy that allows us to perform selective extractions of certain molecules with pharmacological interest. This latter, along with its superior extraction efficiency and reduced environmental and lower economic impacts, make NADESs an interesting alternative to organic solvents for the extraction of *Boldo* bioactive metabolites.

## Figures and Tables

**Table 1:** Alkaloids identified from *P. boldus* with the high-performance liquid chromatography coupled with Ion Trap mass spectrometry (HPLC-IT-MS/MS) method in a positive ionization mode.

Peak *	t_R_ (min)	λ Max (nm)	Identified Compound	[M + H]^+^ *m*/*z*	MS/MS Fragments	Ref.
**6**	15.2	281, 302	coclaurine	286.1	**269.0**, 237.0, 209.0, 175.0, 137.0	[29,30]
**7**	15.8	283, 303	*N*-methylcoclaurine	300.1	**269.1**, 237.1, 209.0, 175.0, 137.0	[29,30,31]
**8**	16.3	282, 302	laurolitsine	314.0	297.1, **265.0**	[32]
**9**	17.3	281, 302	isoboldine	328.1	297.1, **265.0**, 237.1, 165.0	[31,33]
**10**	17.8	280, 303	boldine	328.1	297.1, **265.0**, 237.0, 205.0	[29,34]
**15**	21.0	265, 282	reticuline	330.0	**192.1**, 175.1, 137.0	[31,32,33,34,35,36]
**21**	24.6	266, 303	isocorydine	342.1	296.1, **279.1**, 264.0, 248.1,	[37]
**27**	26.8	283, 302,	laurotetanine	328.1	311.1, **279.1**, 248.1, 219.1, 191.1	[29,32]
**28**	27.5	282, 303	*N*-methyllaurotetanine	342.2	311.1, 296.1, **280.1**, 265.1, 253.1, 237.1	[29,32]

* Peak numbers are the same as the ones depicted in Figure 2. Bold values represent the base peak of the mass spectra.

**Table 2:** Alkaloids identified from *P. boldus* with the high-performance liquid chromatography coupled with quadrupole time-of-flight high resolution mass spectrometry (HPLC QTOF-MS/MS) method in positive ionization mode.

Peak *	t_R_ (min)	Identified Compound	Formula	Mass Experimental	Mass Calculated	Error ppm	[M + H]^+^ *m*/*z*	MS-MS Fragments
**6**	25.64	Coclaurine	C_17_H_19_NO_3_	285.13653	285.13649	0.14	286.14390	**269.11697**, 237.09093, 209.09577, 175.07523
**7**	26.12	*N*-methylcoclaurine	C_18_H_21_NO_3_	299.15228	299.15214	0.47	300.15937	**269.11659**, 237.09041, 209.09544, 175.07485
**8**	26.51	Laurolitsine	C_18_H_19_NO_4_	313.13133	313.13141	0.26	314.13859	**297.11174**, 265.08608, 209.09629, 165.06924
**9**	27.50	Isoboldine	C_19_H_21_NO_4_	327.14718	327.14706	0.38	328.15444	297.11182, **265.08594**, 237.09035, 165.06932
**10**	27.55	Boldine	C_19_H_21_NO_4_	327.14716	327.14706	0.31	328.15445	297.11248, **265.08617**, 237.09124, 205.06469
**15**	27.41	Reticuline	C_19_H_23_NO_4_	329.16323	329.16271	1.6	330.17059	**192.10222**, 239.10637 175.07548, 137.05999
**21**	30.02	Isocorydine	C_20_H_23_NO_4_	341.16282	341.16271	0.32	342.17014	296.10428, **279.10209**, 264.07821, 248.08325
**27**	30.08	Laurotetanine	C_19_H_21_NO_4_	327.14735	327.14706	0.9	328.13587	311.12739, **279.10199**, 248.08321, 191.08531
**28**	32.55	*N*-Methyl-laurotetanine	C_20_H_23_NO_4_	341.16313	341.16271	1.23	342.17048	311.12849, 296.10546, **280.11149**, 265.08649

* Peak numbers are the same as the ones depicted in Figure 2. Bold values represent the base peak of the mass spectra.

**Table 3:** Composition of the Natural Deep Eutectic Solvents (NADES) used in the present study.

Code	Component 1 (HBD)	Component 2 (HDA)	Molar Ratio	Water (%)	Ref.
**NADES1**	Choline chloride	1,2-propanediol	1:3	20%	[25,37,38,39,40,41,42,43]
**NADES2**	Choline chloride	Glycerol	1:2	20%	[25,28,38]
**NADES3**	Choline chloride	Lactic acid	1:2	20%	[25,26,27,28]
**NADES4**	Choline chloride	Levulinic acid	1:1	20%	[25,26,27,28]
**NADES5**	l-Proline	Citric acid	1:2	20%	[25,26,27,28]
**NADES6**	l-Proline	Oxalic acid	1:1	20%	[25,26,27,28]
**NADES7**	l-Proline	Levulinic acid	1:1	20%	[25,26,27,28]

**Table 4:** Bibliographic data on the efficiency of various methodologies regarding boldine recovery from *P. boldus.*

Extraction Method, Analysis	Boldine Yields	Reference
Ethanolic extract, HPLC	0.14%	[10]
European Pharmacopoeia, HPLC-UV	0.016 to 0.059%	[45]
European Pharmacopoeia, HPLC-UV	0.01 to 0.05%	[46]
European Pharmacopoeia, HPLC-UV	0.06%	[47]
European Pharmacopoeia, UHPLC-MS/MS	0.01 to 0.018%	[7]
UAE (water) 23 W/cm2, 36 °C, 40 min	0.148 %	[48]
MAE (water) 200 W, 7.5% S/L, 56 min	0.122%	[49]
NADES6: Proline-oxalic acid, 340 rpm, 50 °C, 50 min, HPLC	0.24%	This work

## Supplementary Materials

The following are available online at https://www.mdpi.com/2223-7747/9/2/242/s1, Table S1: HPLC IT-MS and MS/MS data for *P. boldus* phenolics compounds and their proposed structures, Table S2: HPLC QTOF-MS for the phenolics compounds of *P. boldus* and their proposed structures, Figure S1: MS-MS spectra of *P. boldus* alkaloids. The spectra correspond to: (a) coclaurine, (b) *N*-methylcoclaurine, (c) laurolitsine, (d) isoboldine, (e) boldine, (f) reticuline, (g) isocoridine,(h) laurotetanine (i) *N*-methyllaurotetanine, Figure S2: HPLC-PDA-QTOF-MS of *P. boldus*. UV trace is at 280 nm. Figure S3: QTOF MS/MS spectra of *P. boldus* alkaloids. The spectra correspond to: (a) coclaurine, (b) *N*-methylcoclaurine, (c) laurolitsine, (d) isoboldine, (e) boldine, (f) reticuline, (g) isocoridine, (h) laurotetanine (i) *N*-methyllaurotetanine. Figure S4: General scheme of fragmentation for isoquinoline alkaloids identified in *P. boldus*. Numbers between round brackets correspond to the alkaloids listed in Table 1 and Table 2 in the main manuscript. Figure S5: General scheme of fragmentation for aporphine alkaloids identified in *P. boldus*. Numbers between round brackets correspond to the alkaloids listed in Table 1 and Table 2 in main manuscript.

## References

[1] Fernández J., Lagos P., Rivera P., Zamorano-Ponce E. (2009). Effect of boldo (*Peumus boldus* Molina) infusion on lipoperoxidation induced by cisplatin in mice liver. Phytother. Res..

[2] Looser G. (1935). ¿Cuál es el verdadero nombre botánico del peumo y del boldo?. Rev. Chil. Hist. Nat..

[3] Speisky H., Squella J.A., Núñez-Vergara L.J. (1991). Activity of boldine on rat ileum. Planta Med..

[4] Speisky H., Cassels B. (1994). Boldo and boldine: An emerging case of natural drug development. Pharmacol. Res..

[5] O’Brien P., Carrasco-Pozo C., Speisky H. (2006). Boldine and its antioxidant or health-promoting properties. Chem. Biol. Interact..

[6] Simirgiotis M.J., Schmeda-Hirschmann G. (2010). Direct identification of phenolic constituents in boldo Folium (*Peumus boldus* Mol.) infusions by high-performance liquid chromatography with diode array detection and electrospray ionization tandem mass spectrometry. J. Chromatogr. A.

[7] Fuentes-Barros G., Castro-Saavedra S., Liberona L., Acevedo-Fuentes W., Tirapegui C., Mattar C., Cassels B. (2018). Variation of the alkaloid content of *Peumus boldus* (boldo). Fitoterapia.

[8] Bradley P.R. (2006). British Herbal Compendium: A Handbook of Scientific Information on Widely Used Plant Drugs.

[9] EMA-HMPC Community Herbal Monograph on *Peumus boldus* Molina, Folium. https://www.ema.europa.eu/en/documents/herbal-opinion/opinion-hmpc-european-union-herbal-monograph-peumus-boldus-molina-folium_en.pdf.

[10] Schmeda-Hirschmann G., Rodríguez J.A., Theoduloz C., Astudillo S.L., Feresin G.E., Tapia A. (2003). Free-radical Scavengers and Antioxidants from *Peumus boldus* Mol. (“Boldo”). Free Radic. Res..

[11] Quezada M., Asencio M., Valle J.M., Aguilera J.M., Gomez B. (2004). Antioxidant activity of crude extract, alkaloid fraction and flavonoid fraction from boldo (*Peumus boldus* Molina) leaves. J. Food Sci..

[12] Cermanova J., Kadova Z., Zagorova M., Hroch M., Tomsik P., Nachtigal P., Kudlackova Z., Pavek P., Dubecka M., Ceckova M. (2015). Boldine enhances bile production in rats via osmotic and Farnesoid X receptor dependent mechanisms. Toxicol. Appl. Pharmacol..

[13] Gomez G., Velarde V. (2018). Boldine Improves Kidney Damage in the Goldblatt 2K1C Model Avoiding the Increase in TGF-β. Int. J. Mol. Sci..

[14] Konrath E., Santin K., Nassif M., Latini A., Henriques A., Salbego C. (2008). Antioxidant and pro-oxidant properties of boldine on hippocampal slices exposed to oxygen-glucose deprivation in vitro. Neurotoxicology.

[15] Schirckel S., Bittner M. (2010). La salud en nuestras manos: Plantas medicinales en Chile, Riqueza Natural y Científica.

[16] Soto C., Caballero E., Pérez E., Zúniga M. (2013). Effect of extraction conditions on total phenolic content and antioxidant capacity of pretreated wild *Peumus boldus* leaves from Chile. Food Bioprod. Process..

[17] Lu P., Sun H., Zhang L., Hu H., Zhang L., Zhao F., Ge C., Yao M., Wang T., Li J. (2012). Isocorydine targets the drug-resistant cellular side population through PDCD4-related apoptosis in hepatocellular carcinoma. Mol. Med..

[18] Pastene E., Parada V., Avello M., Ruiz A., García A. (2014). Catechin-based Procyanidins from *Peumus boldus* Mol. Aqueous Extract Inhibit Helicobacter pylori Urease and Adherence to Adenocarcinoma Gastric Cells. Phytother. Res..

[19] Abbott A.P., Boothby D., Capper G., Davies D.L., Rasheed R.K. (2004). Deep Eutectic Solvents Formed between Choline Chloride and Carboxylic Acids: Versatile Alternatives to Ionic Liquids. J. Am. Chem. Soc..

[20] Zhang Q., De Oliveira Vigier K., Royer S., Jerome F. (2012). Deep eutectic solvents: Syntheses, properties and applications. Chem. Soc. Rev..

[21] Smith E.L., Abbott A.P., Ryder K.S. (2014). Deep Eutectic Solvents (DESs) and Their Applications. Chem. Rev..

[22] Abidin Z., Hamdi M., Maan H., Adeeb H., Subramanian J.N. (2017). New horizons in the extraction of bioactive compounds using deep eutectic solvents: A review. Anal. Chim. Acta.

[23] Choi Y.H., van Spronsen J., Dai Y., Verberne M., Hollmann F., Arends I.W.C.E., Witkamp G.J., Verpoorte R. (2011). Are Natural Deep Eutectic Solvents the Missing Link in Understanding Cellular Metabolism and Physiology?. Plant Physiol..

[24] Liu Y., Friesen J.B., McAlpine J.B., Lankin D.C., Chen S.N., Pauli G.F. (2018). Natural Deep Eutectic Solvents: Properties, Applications, and Perspectives. J. Nat. Prod..

[25] Dai Y., van Spronsen J., Witkamp G.J., Verpoorte R., Choi Y.H. (2013). Natural deep eutectic solvents as new potential media for green technology. Anal. Chim. Acta.

[26] Jiang Z.M., Wang L.W., Gao Z., Zhuang B., Yin Q., Liu E.H. (2019). Green and efficient extraction of different types of bioactive alkaloids using deep eutectic solvents. Microchem. J..

[27] Takla S.S., Shawky E., Hammoda H., Darwish F. (2018). Green techniques in comparison to conventional ones in the extraction of Amaryllidaceae alkaloids: Best solvents selection and parameters optimization. J. Chromatogr. A.

[28] Shawky E., Takla S.S., Hammoda H., Darwish F. (2018). Evaluation of the influence of green extraction solvents on the cytotoxic T activities of Crinum (Amaryllidaceae) alkaloid extracts using in-vitro-in-silico approach. J. Ethnopharmacol..

[29] He Y., Cheng P., Wang W., Yan S., Tang Q., Liu D., Xie H. (2018). Rapid Investigation and Screening of Bioactive Components in Simo Decoction via LC-Q-TOF-MS and UF-HPLC-MD Methods. Molecules.

[30] Sharma B., Yadav A., Dabur R. (2019). Interactions of a medicinal climber *Tinospora cordifolia* with supportive interspecific plants trigger the modulation in its secondary metabolic profiles. Sci. Rep..

[31] Bakiri A., Hubert J., Reynaud R., Lanthony S., Harakat D., Renault J.H., Nuzillard J.M. (2017). Computer-Aided 13C NMR Chemical Profiling of Crude Natural Extracts without Fractionation. J. Nat. Prod..

[32] Soares E.R., da Silva F.M., de Almeida R.A., de Lima B.R., da Silva Filho F.A., Barison A., Koolen H.H., Pinheiro M.L.B., de Souza A.D. (2015). Direct infusion ESI-IT-MSn alkaloid profile and isolation of tetrahydroharman and other alkaloids from *Bocageopsis pleiosperma* maas (Annonaceae). Phytochem. Anal..

[33] Nikolic D., Gödecke T., Chen S.N., White J., Lankin D., Pauli G.F., Van Breemen R. (2012). Mass spectrometric dereplication of nitrogen-containing constituents of black cohosh (*Cimicifuga racemosa* L.). Fitoterapia.

[34] Singh A., Bajpai V., Kumar S., Singh A.K., Kumar B. (2017). Analysis of isoquinoline alkaloids from *Mahonia leschenaultia* and *Mahonia napaulensis* roots using UHPLC-Orbitrap-MSn and UHPLC-QqQLIT-MS/MS. J. Pharm. Anal..

[35] Del Mar-Contreras M., Noureddine B., Gómez-Caravaca A., Gálvez J., Segura-Carretero A. (2017). Alkaloids Profiling of *Fumaria capreolata* by Analytical Platforms Based on the Hyphenation of Gas Chromatography and Liquid Chromatography with Quadrupole-Time-of-Flight Mass Spectrometry. Int. J. Anal. Chem..

[36] De Lima B.R., da Silva F.M.A., Soares E.R., de Almeida R.A., da Silva-Filho F.A., Barison A., Costa E.V., Koolen H.H.F., de Souza A.D.L., Pinheiro M.L.B. (2020). Integrative Approach Based on Leaf Spray Mass Spectrometry, HPLC-DAD-MS/MS, and NMR for Comprehensive Characterization of Isoquinoline-Derived Alkaloids in Leaves of *Onychopetalum amazonicum* R. E. Fr. J. Braz. Chem. Soc..

[37] Duan L., Dou L.L., Guo L., Li P., Liu E.H. (2016). Comprehensive evaluation of deep eutectic solvents in extraction of bioactive natural products. ACS Sustain. Chem. Eng..

[38] Bajkacz S., Adamek J. (2017). Evaluation of new natural deep eutectic solvents for the extraction of isoflavones from soy products. Talanta.

[39] Katsampa P., Valsamedo E., Grigorakis S., Makris D. (2015). A green ultrasound-assisted extraction process for the recovery of antioxidant polyphenols and pigments from onion solid wastes using Box–Behnken experimental design and kinetics. Ind. Crops Prod..

[40] Ruesgas-Ramón M., Figueroa-Espinoza M.C., Durand E. (2017). Application of Deep Eutectic Solvents (DES) for Phenolic Compounds Extraction: Overview, Challenges, and Opportunities. J. Agric. Food Chem..

[41] González C., Mustafa N., Wilson E., Verpoorte R., Choi Y. (2018). Application of natural deep eutectic solvents for the “green” extraction of vanillin from vanilla pods. Flavour Fragr. J..

[42] Savi L., Carpiné D., Waszczynskyj N., Ribani R., Haminiuk C. (2019). Influence of temperature, water content and type of organic acid on the formation, stability and properties of functional natural deep eutectic solvents. Fluid Phase Equilibria.

[43] Mulia K., Krisanti E., Terahadi F., Putri S. (2015). Selected Natural Deep Eutectic Solvents for the Extraction of α-Mangostin from Mangosteen (*Garcinia mangostana* L.) Pericarp. Int. J. Technol..

[44] Rogalinski T., del Valle J.M., Zetzl C., Brunner G. (2003). Extraction of boldo (*Peumus boldus* Mol.) leaves with hot pressurized water and supercritical CO_2_. Food Res. Int..

[45] Schwanz M. (2006). Desenvolvimento e validacão de método analiítico para quantificacão da boldina em *Peumus boldus* Mol. (Monimiaceae) e avaliacão preliminar de sua estabilidade. Ph.D. Thesis.

[46] Espic M. (2007). Evaluación de la producción de biomasa aérea y del rendimiento en aceite esencial y boldina, en boldo (Peumus boldus Mol.) en la comuna de Papudo V región. Bachelor’s Thesis.

[47] Camara C.I., Bornancini C.A., Cabrera J.L., Ortega M.G., Yudi L.M. (2010). Quantitative analysis of boldine alkaloid in natural extracts by cyclic voltammetry at a liquid–liquid interface and validation of the method by comparison with high performance liquid chromatography. Talanta.

[48] Petigny L., Périno-Issartier S., Wajsman J., Chemat F. (2013). Batch and Continuous Ultrasound Assisted Extraction of Boldo Leaves (*Peumus boldus* Mol.). Int. J. Mol. Sci..

[49] Petigny L., Périno S., Minuti M., Visinoni F., Wajsman J., Chemat F. (2014). Simultaneous Microwave Extraction and Separation of Volatile and Non-Volatile Organic Compounds of Boldo Leaves. From Lab to Industrial Scale. Int. J. Mol. Sci..

[50] Gómez A.V., Tadini C.C., Biswas A., Buttrum M., Kim S., Boddu V.M., Cheng H.N. (2019). Microwave-assisted extraction of soluble sugars from banana puree with natural deep eutectic solvents (NADES). LWT.

[51] Si Y.Y., Sun S.W., Liu K., Liu Y., Shi H.L., Zhao K., Wang J., Wang W. (2019). Novel Deep Eutectic Solvent Based on Levulinic Acid and 1,4-Butanediol as an Extraction Media for Bioactive Alkaloid Rutaecarpine. Processes.

[52] Liu Y., Garzon J., Friesen J.B., Zhang Y., McAlpine J.B., Lankin D.C., Chen S.-N., Pauli G.F. (2016). Countercurrent assisted quantitative recovery of metabolites from plant-associated natural deep eutectic solvents. Fitoterapia.

[53] Smink D., Kersten S.R.A., Schuur B. (2020). Recovery of lignin from deep eutectic solvents by liquid-liquid extraction. Sep. Purif. Technol..

[54] Kim K.H., Dutta T., Sun J., Simmons N., Singh S. (2018). Biomass pretreatment using deep eutectic solvents from lignin derived phenols. Green Chem..

[55] Liang X., Fu Y., Chang J. (2019). Effective separation, recovery and recycling of deep eutectic solvent after biomass fractionation with membrane-based methodology. Sep. Purif. Technol..

[56] Sanap A.K., Shankarling G.S. (2014). Eco-Friendly and recyclable media for rapid synthesis of tricyanovinylated aromatics using biocatalyst and deep eutectic solvent. Catal. Commun..

[57] Phadtare S.B., Shankarling G.S. (2010). Halogenation reactions in biodegradable solvent: Efficient bromination of substituted 1-aminoanthra-9, 10-quinone in deep eutectic solvent (choline chloride: Urea). Green Chem..

[58] Singh B.S., Lobo H.R., Pinjari D.V., Jarag K.J., Pandit A.B., Shankarling G.S. (2013). Ultrasound and deep eutectic solvent (DES): A novel blend of techniques for rapid and energy efficient synthesis of oxazoles. Ultrason. Sonochem..

[59] Singleton V.L., Rossi J.A. (1965). Colorimetry of total phenolics with phosphomolibdic phosphotungstic acid reagent. Am. J. Enol. Vitic..

[60] Bordiga M., Gómez-Alonso S., Locatelli M., Travaglia F., Coïsson J.D., Hermosín-Gutiérrez I., Arlorio M. (2014). Phenolics characterization and antioxidant activity of six different pigmented *Oryza sativa* L. cultivars grown in Piedmont (Italy). Food Res. Int..

[61] (2002). Directive 2002/657/EC. Commission Decision of 12 August 2002 Implementing Council Directive 96/23/EC Concerning the Performance of Analytical Methods and the Interpretation of Results (Notified under Document Number C(2002) 3044).

