# Supplementary material for: Green Extraction of Alkaloids and Polyphenols from Peumus boldus Leaves with Natural Deep Eutectic Solvents and Profiling by HPLC-PDA-IT-MS/MS and HPLC-QTOF-MS/MS
Source: Plants (Basel). 2020 Feb 13;9(2):242. doi: 10.3390/plants9020242 (PMC7076633; doi:10.3390/plants9020242)
Supplement: Supplementary file 1 [file plants-09-00242-s001.pdf]

SUPPLEMENTARY MATERIALS

Table S1. HPLC IT-MS and MS/MS data for *P. boldus* phenolics compounds and their proposed structures.

Nº Peak	t_R (min)	$[M+H]^+$ *m/z*	MS/MS fragments	λ Max (nm)	Identified compound
1	3.1	579.1	560.9, 453.0, **427.0**, **409.0**, 291.0, 247.0, 163.0	280	procyanidin dimer
2	3.5	579.1	561.1, 453.1, 427.0, **409.0**, 291.0, 246.9	280	procyanidin dimer
3	4.3	290.4	272.0, 244.0, **226.0**, 208.0, 123.0	279	Catechin
4	5.1	291.1	273.0, 165.0, 139.0, 123.1	280	Epicatechin
5	8.4	579.2	561.0, 525.1, 453.0, 427.0, **409.0**, 332.9, 300.9, 291.0	280	procyanidin dimer
11	19.0	727.2	595.0**, 433.0,** 287.0	267, 288, 349	luteolin-pentosyl glucosylrhamnose
12	19.8	611.1	**449.0**, 303.0	263, 289 sh, 354	heperidin-7-O-rhamnoglucoside
13	20.6	757.2	625.0, **463.0,** 317.0	263, 290 sh, 354	myricetin-rhamnosyl-glucosyl pentoside
14	20.9	581.1	449.0, 303.0	264, 290, 353	quercetin pentosyl-rhamnoside
16	21.6	595.1	433.0, 287.0	264,291 sh, 352	luteolin 3-O-rutinoside
17	22.1	697.2	565.0**, 433.0**, 419.0, 383.0, 353.0, 287.0	264, 292 sh, 348	luteolin dipentosyl rhamnoside
18	23.1	595.1	433.0, 287.0	270, 284, 351	luteolin 7-O-rutinoside
19	23.4	625.2	463.0, 316.9	266, 288 sh, 351	myricetin rhamnosyl-hexose
20	23.8	727.1	595.0, 463.0, 413.0, 317.0	265, 289, 352	myricetin pentosyl-hexosyl-rhamnoside
22	24.9	771.2	625.0, 463.0, 317.0	264, 290 sh, 349	isorhamnetin rhamnosyl-glucosyl-rhamnoside
23	25.1	286.2	269.0, 237.0, 175.0, 143.0	264, 282 sh, 349	Luteolin
24	25.6	595.2	463.0, 317.0	265, 288 sh, 353	myricetin rhamnosyl-pentoside
25	25.8	595.1	463.0, 317.0, 287.0	266, 289 sh, 346	luteolin glycoside
26	26.4	579.1	433.0, 287.0	265, 315,344	luteolin-dirhamnoside
29	27.7	609.1	463.0, 317.0	264, 317, 348	myricetin dirhamnoside
30	28.0	741.2	609.1, 595.0, **463.0**, 445.0, 317.0	267, 348	kaempferol-3-O-glucosyl-rhamnosyl-rhamnose
31	43.2	595.1	308.9, **286.9**	268, 304 sh, 314	kaempferol 3-O-coumaroyl-glucoside

Table S2. HPLC QTOF-MS for the phenolics compounds of *P. boldus* and their proposed structures.

Peak*	t_R (min)	Formula	Mass experimental	Mass Calculated	Error ppm	Identified compounds
1	3.1	$C_{30}H_{26}O_{12}$	578.14142	578.14243	1.74	procyanidin dimer
2	3.5	$C_{30}H_{26}O_{12}$	578.14152	578.14273	1.21	procyanidin dimer
	4.3	$C_{15}H_{14}O_6$	290.07839	290.07904	2.21	catechin
4	5.1	$C_{15}H_{14}O_6$	290.07839	290.07904	2.24	Epicatechin
13	20.6	$C_{33}H_{40}O_{20}$	756.20935	756.21129	2.57	myricetin-rhamnosyl-glucosyl pentoside
17	22.1	$C_{34}H_{32}O_{16}$	696.17072	696.16903	1.68	luteolin dipentosyl rhamnoside
19	23.4	$C_{28}H_{32}O_{16}$	624.16833	624.16903	1.14	myricetin rhamnosyl-hexose
24	25.6	$C_{30}H_{26}O_{13}$	594.13636	594.13734	1.67	myricetin rhamnosyl-pentoside
30	28.0	$C_{33}H_{40}O_{19}$	740.21449	740.21638	2.55	kaempferol-3-O-glucosyl-rhamnosyl-rhamnose

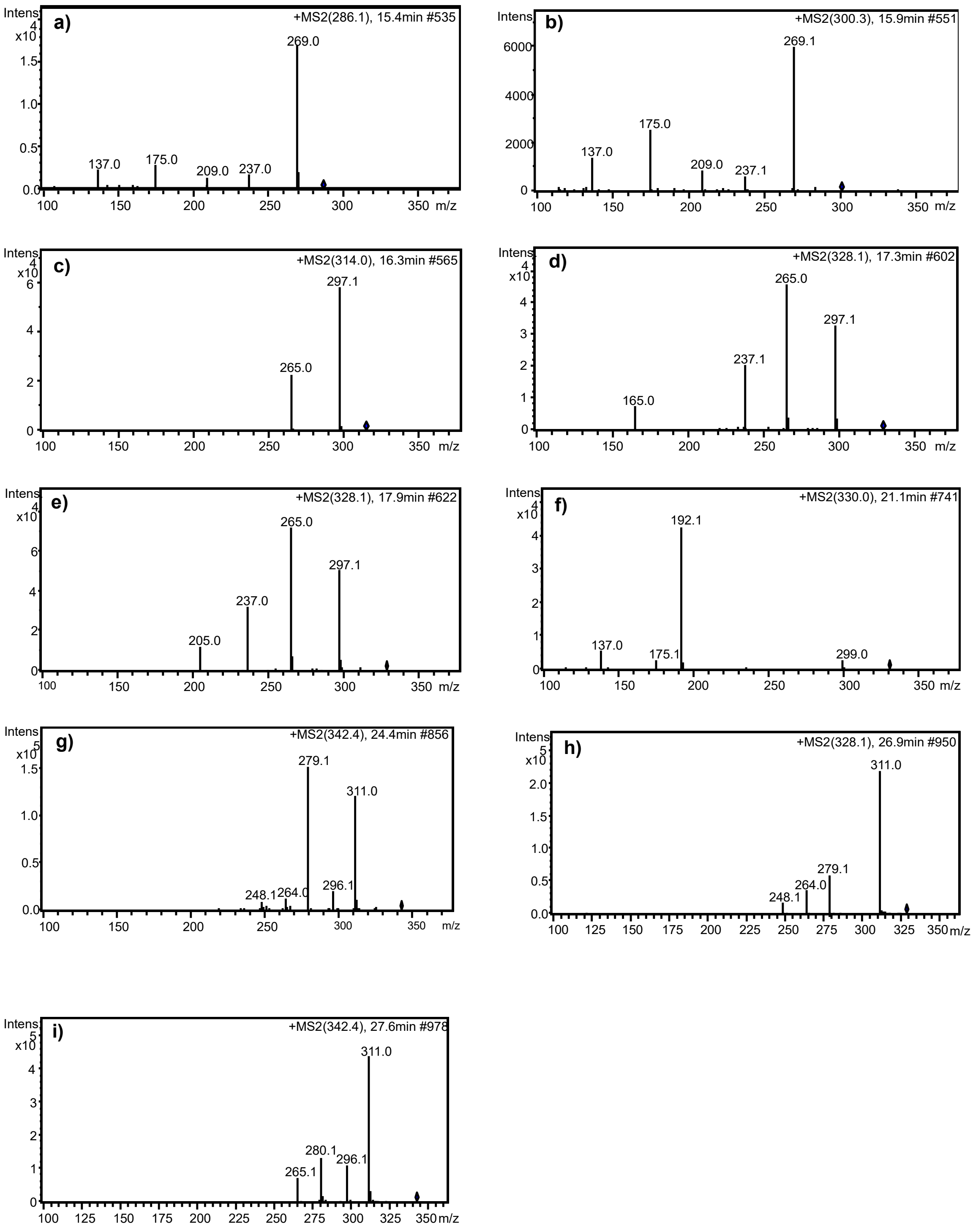

Figure S1. MS-MS spectra of *P. boldus* alkaloids. The spectra correspond to: (a) coclaurine, (b) *N*-methylcoclaurine, (c) laurolitsine, (d) isoboldine, (e) boldine, (f) reticuline, (g) isocoridine, (h) laurotetanine (i) *N*-methyllaurotetanine.

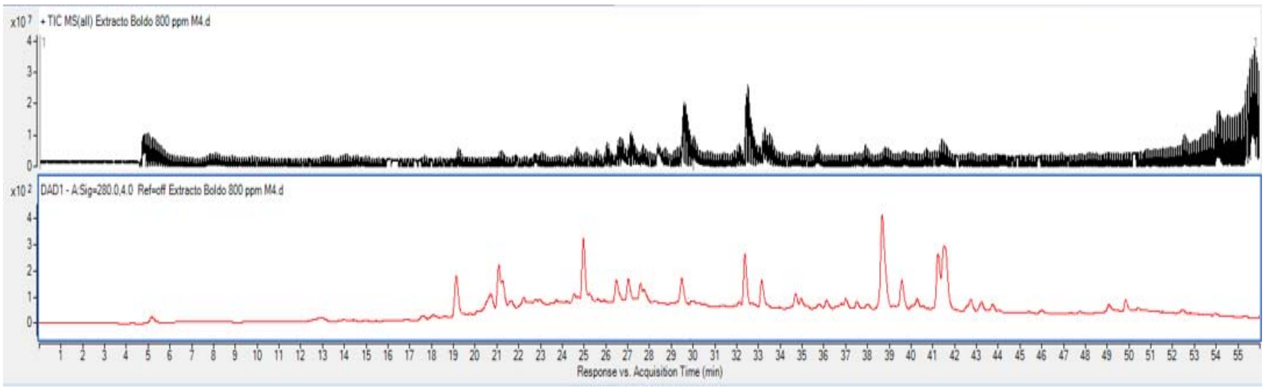

Figure S2. HPLC-PDA-QTOF-MS of *P. boldus*. UV trace is at 280 nm.

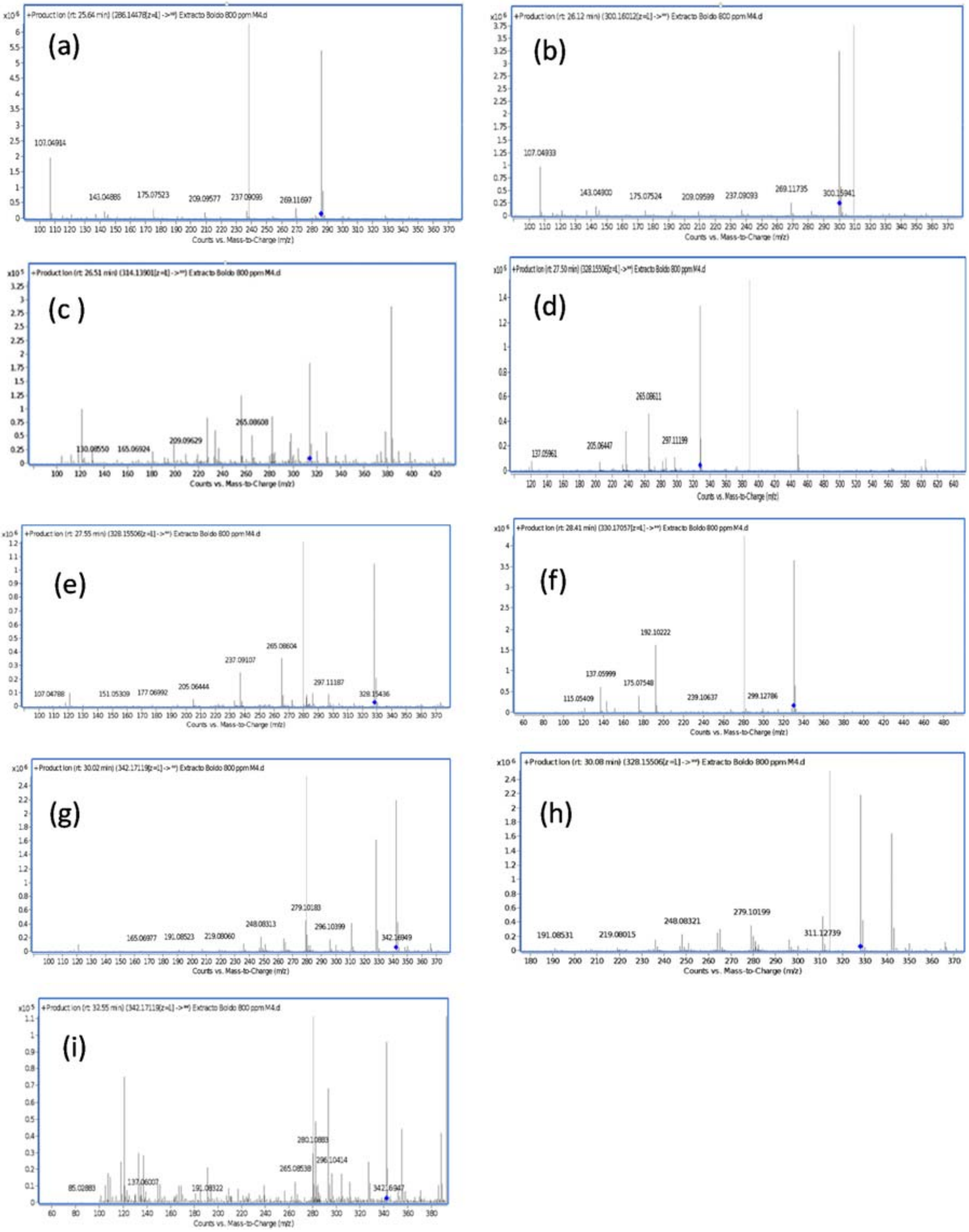

Figure S3. QTOF MS/MS spectra of *P. boldus* alkaloids. The spectra correspond to: (a) coclaurine, (b) *N*-methylcoclaurine, (c) laurolitsine, (d) isoboldine, (e) boldine, (f) reticuline, (g) isocoridine, (h) laurotetanine (i) *N*-methyllaurotetanine

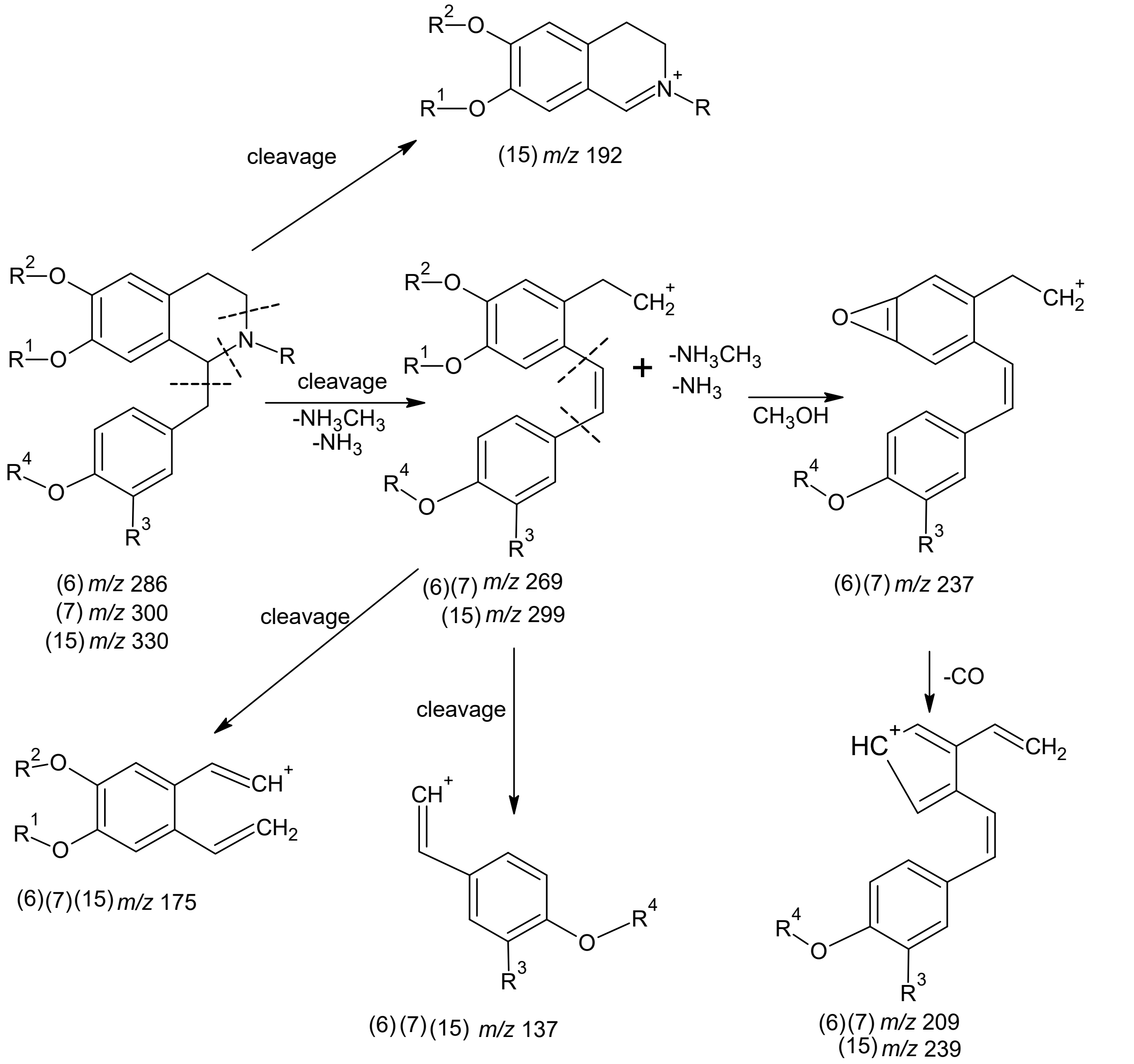

Figure S4. General scheme of fragmentation for isoquinoline alkaloids identified in *P. boldus*. Numbers between round brackets correspond to the alkaloids listed in Table 1 and 2 in the main manuscript.

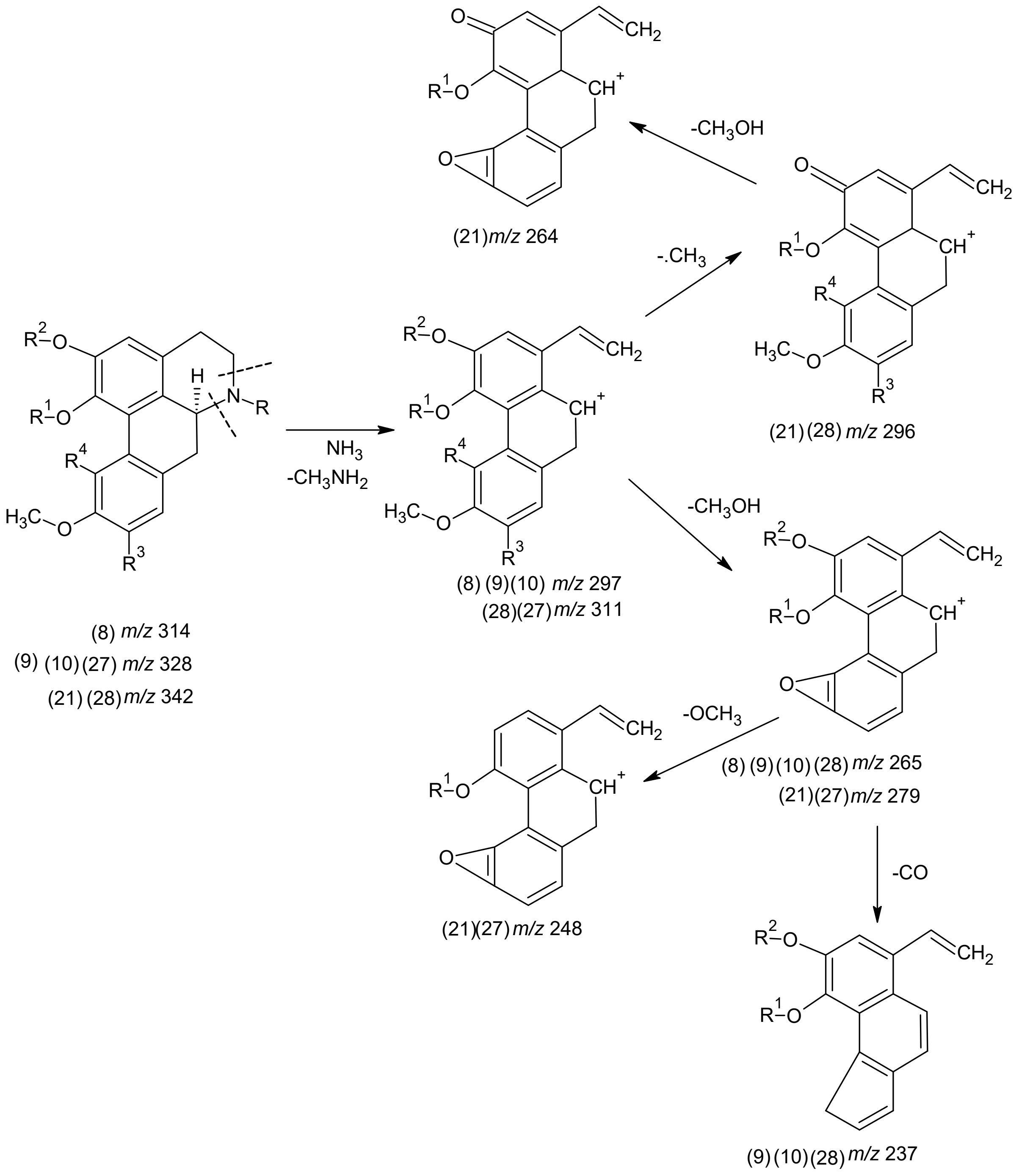

Figure S5. General scheme of fragmentation for aporphine alkaloids identified in *P. boldus*. Numbers between round brackets correspond to the alkaloids listed in Table 1 and 2 in the main manuscript.
